# Supplementary material for: Preclinical Development of FA5, a Novel AMP-Activated Protein Kinase (AMPK) Activator as an Innovative Drug for the Management of Bowel Inflammation
Source: Int J Mol Sci. 2021 Jun 13;22(12):6325. doi: 10.3390/ijms22126325 (PMC8231528; doi:10.3390/ijms22126325)
Supplement: Supplementary file 1 [file ijms-22-06325-s001.zip › Supplemental Figures.pdf]

A

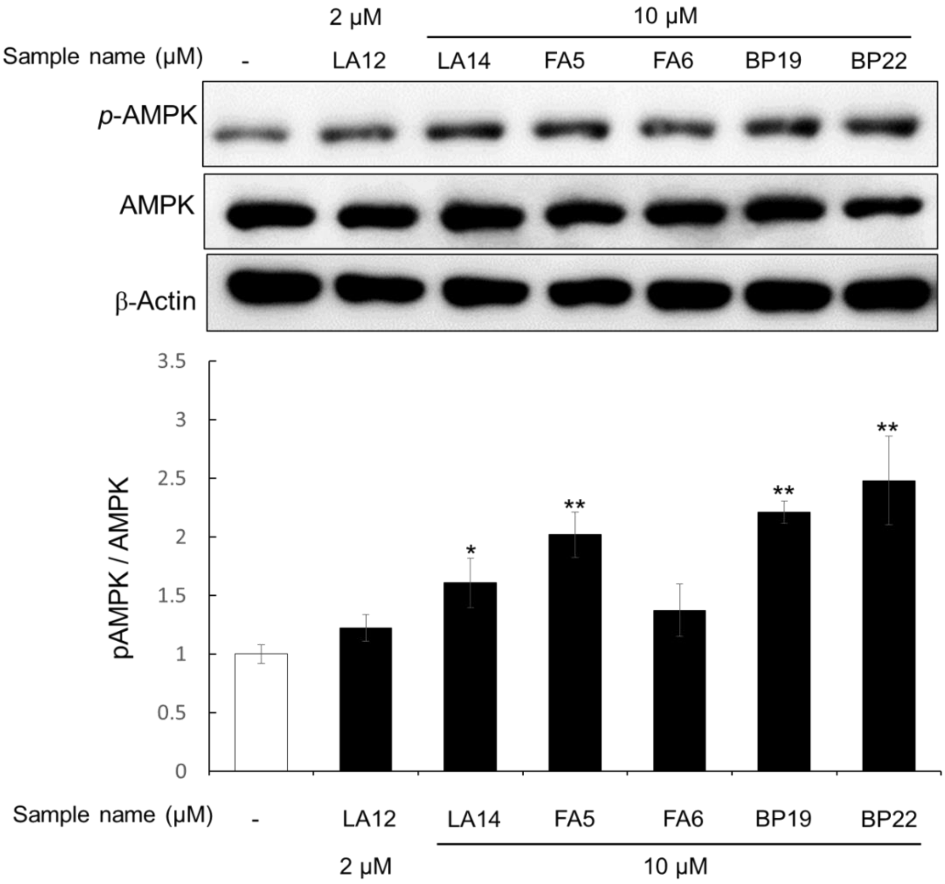

B

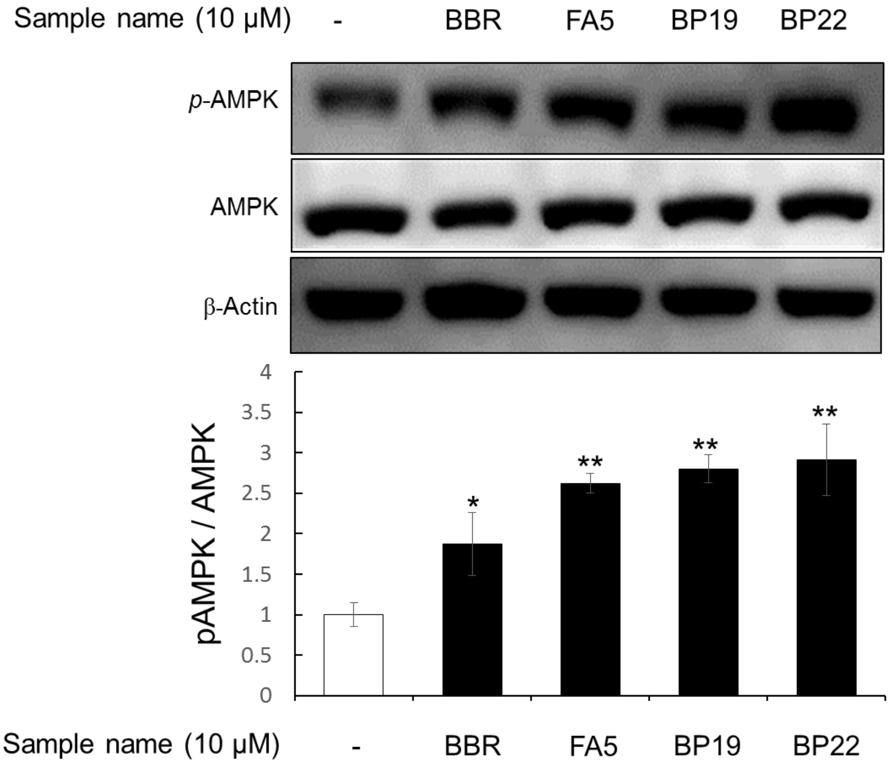

Supplemental Figure S1

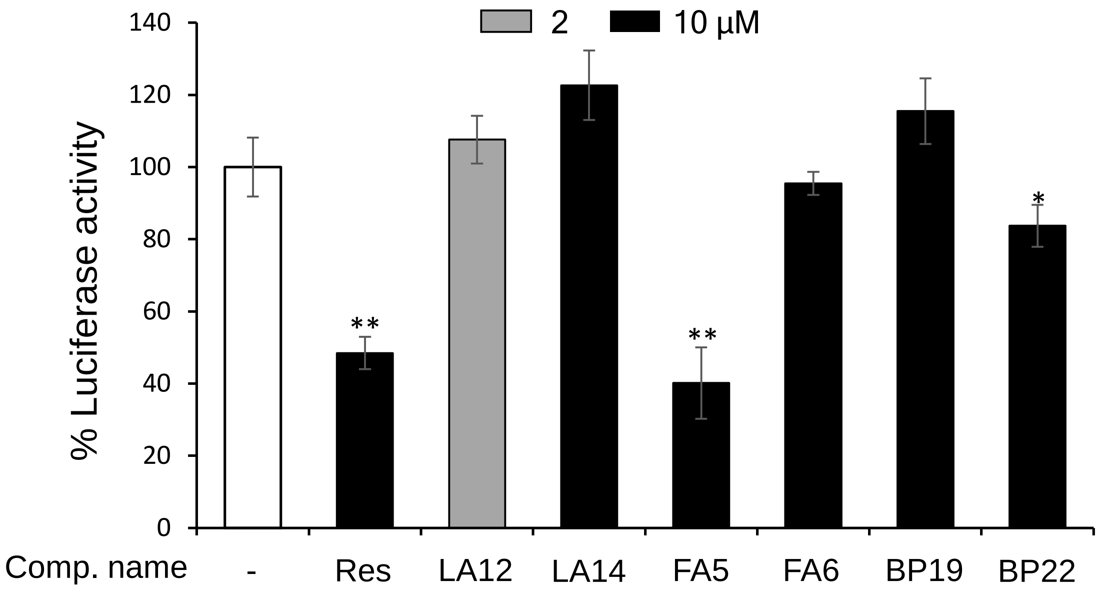

Supplemental Figure S2

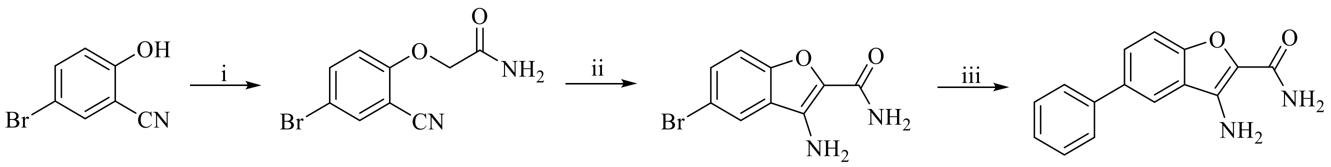

i) Bromoacetamide, Cs_2CO_3, DMF; ii) KOH, EtOH; iii) Phenylboronic Acid, $Pd(OAc)_2$, PPh_3, Toluene.

Supplemental Figure S3
